# Comparing Warming Methods in the Trauma Bay: A Multi-center Cohort Study

**DOI:** 10.7759/cureus.104783

**Published:** 2026-03-06

**Authors:** Gayla Miles, Kristi Bonny, Jacob Roden-Foreman

**Affiliations:** 1 Trauma, Texas Health Harris Methodist Hospital Fort Worth, Fort Worth, USA; 2 Emergency Medicine, Texas Health Presbyterian Hospital Plano, Plano, USA; 3 Trauma, Texas Health Presbyterian Hospital Dallas, Dallas, USA

**Keywords:** cold stress, emergency department, hypothermia, trauma, underbody warming mattress

## Abstract

Background

Despite the clear link between hypothermia and poor outcomes, few studies have evaluated warming methods in the trauma bay. This study aimed to compare the effectiveness of three warming methods for full trauma activations arriving at the emergency department.

Methods

A multi-site, retrospective cohort study was conducted at two Level I and one Level II trauma centers in North Texas, United States, from August 1, 2023, to July 31, 2024. Body temperature data were collected for three warming methods: blankets, forced air underbody warming mattress, or forced air warming mattress plus thermo-reflective head cap. The ability of warming devices to treat/prevent cold stress was tested with Cox hazard models that controlled for known confounders. As study sites did not share standardized warming device workflows or temperature measurement protocols, nesting of patients within hospitals and potential confounding effects of the study sites were addressed with cluster robust standard errors.

Results

Patients were treated with blankets (n = 99), mattresses (n = 92), or mattresses + caps (n = 19). Cap use was rare due to prevalent contraindications. For patients arriving normothermic (n = 98; arrival temperature 36.8 ^o^C ± 0.3), Cox modelling indicated the mattress was not reliably better than blankets at preventing new cold stress (<36.5 ^o^C; p = 0.343). For patients arriving with cold stress (n = 112; arrival temperature 35.7 ^o^C ± 0.9), Cox modelling revealed normothermia was 4.7 times as likely to be achieved with the mattress than with blankets after controlling for confounders (p <0.001).

Conclusion

Application of the forced air underbody warming mattress was feasible and practical in the trauma bay and more effective than blankets in correcting cold stress. The thermally reflective head cap was impractical due to the prevalence of head injuries and the routine need for computerized tomography scans of the head.

## Introduction

An injury severity score (ISS) ≥ 15 is the predominant definition for major trauma and is a universally accepted measure of major trauma based on injury types, body area, and aggregation of injuries [1]. According to the American College of Surgeons Advanced Trauma Life Support (ATLS) 2024 guidelines, seriously injured patients arriving at the emergency department (ED) must be assessed immediately upon arrival to identify the risk of impairment [2]. Upon arrival, hypothermia occurrence in ED trauma bays is common for several reasons: (i) environmental exposure in cold climates, (ii) cool ambient ambulance temperatures in summer months, (iii) injury assessment requiring clothing removal, and (iv) trunk exposure for medical interventions such as chest tube insertions [3]. Hypoperfusion and reduced metabolic heat production from sustained blood loss can further exacerbate these factors [4, 5]. 

While the human body attempts to maintain a core temperature at or near 37°C [6], the occurrence of cold stress and potential shivering occurs when body temperature drops below 36.5°C. The cold stress state is commonly seen in the trauma bays when the patient is uncovered for assessment and procedures. As the temperature drops, hypothermia occurs when core body temperature is <35° C [7]. The goal of clinicians is to identify the process of cold stress and intervene to prevent the state of hypothermia.

A basic intervention to warm the patient and prevent hypothermia in the trauma bay is the application of blankets. However, care providers commonly remove the blankets while trying to work quickly and intensely to complete procedures and assess/repair injuries. During this period, the frequent uncovering can expose up to 90% of the body and lead to rapid heat loss. When a large portion of the body surface area is uncovered in a cool environment, the risk of hypothermia significantly increases due to enhanced heat loss through radiation, conduction, and convection [8]. 

Warmed intravenous (IV) fluids are also commonly used in the trauma bay and the prehospital setting [9,10]. In-line fluid warmers are particularly effective at preventing new-onset hypothermia or cold stress by allowing clinicians to transfuse products at normothermic temperatures instead of at room or refrigerator temperatures; however, the ability of fluid warmers to treat existing hypothermia (<35°C) or cold stress (<36.5°C) is limited and can require using dangerously high temperatures or very high fluid volumes to achieve rewarming goals [11]. This duality highlights one of the difficulties of achieving successful thermal management in trauma: ideal warming methods should be able to keep warm patients warm (prevention) as well as increase the temperatures of initially cold patients (treatment). The effectiveness of in-line warmers in the trauma bay is also limited by the fact that the long IV tubing necessary to avoid obstructing access to the patient produces heat loss [12]. As such, there is a need for additional warming methods that are practical and effective in the trauma bay.

Two warming devices that may have utility in the trauma bay are forced air underbody warming mattresses and thermal head caps. Underbody warming mattresses are placed between patients and the bed and connected to blowers that circulate heated air through flexible tubes embedded in the mattress; this provides continuous, active heat transfer. In contrast, thermal head caps are flexible, metallic head coverings that provide passive heat capture by reducing convective and radiant heat loss from the head. Many surgical areas now use these devices, which can prevent up to 64% heat loss [13-15]. Despite the success of these devices in the perioperative area, their use as adjuncts or replacements for other warming methods (e.g., warmed fluids) has not been widely studied in trauma bays.

This study aimed to assess and compare the effectiveness of forced air underbody warming mattresses with or without thermal head caps versus standard warmed blankets in the trauma bay. Notably, the head cap was not evaluated as an independent intervention but only as an adjunct to the underbody warming mattress due to feasibility constraints. The primary aim was to compare the ability of these devices to achieve overall successful thermal management (i.e., treatment and prevention of cold stress). Secondary aims included separately comparing the treatment and prevention of cold stress between devices. 

## Materials and methods

This was a multisite retrospective cohort study conducted at three urban American College of Surgeons (ACS)-verified trauma centers (two Level I, Texas Health Harris Methodist Hospital Fort Worth and Texas Health Presbyterian Hospital Dallas, and one Level II, Texas Health Presbyterian Hospital Plano) in north Texas. The study was approved by the Human Research Protection Program Office, Texas Health Resources (approval number STU -2023-0305). The STROBE (Strengthening the Reporting of Observational Studies in Epidemiology) guidelines were utilized for reporting.

Sample size calculation

Sample size analysis was conducted using G*Power version 3.1 (Heinrich-Heine University, Düsseldorf, Germany) and indicated that 130 patients per group would be needed to achieve 80% power to detect a change from 75% to 90% treatment effectiveness at alpha = 0.05 using Fisher’s exact test with Bonferroni correction for pairwise comparisons [16,17]. Due to resource limitations across the three sites, the protocol called for collecting data until the required sample was achieved or for up to 12 months. Each site used its institutional trauma registries to periodically screen recent patients for eligibility and retrospectively collect data from electronic medical records for one year.

Comparison groups and warming devices

This study compared the relative effectiveness of three warming devices. All three sites had cotton blankets stored in warming cabinets (Blickman Blanket Warmer; Blickman Industries, Lyndhurst, New Jersey, United States, and Pedigo Blanket Warmer; Pedigo Products, Inc., Vancouver, Washington, United States) set to 51.7-54.4°C in the trauma bay. The underbody warming mattress was the Bair Hugger™ Full Access Underbody Blanket, Model 635 (3M Company, Maplewood, Minnesota, United States). The underbody mattress was attached to the warming unit (Bair Hugger™ Warming Unit, Model 77500; 3M Company), which was placed on the medium setting (38°C) for all patients. The thermally reflective head cap was the Thermoflect Adult Bouffant Cap, Model 5110-100 (Encompass Group, LLC, McDonough, Georgia, United States). The cap was only used as an adjunct to the underbody mattress and was not evaluated independently. The cap was applied only if cleared by a participating neurosurgeon and was determined acceptable for use only if no obvious injury occurred to the head.

Eligibility criteria

The study included patients arriving to participating sites between August 2023 and July 2024 and met the following inclusion criteria: aged ≥18 years, received a full trauma team activation, had an initial ED temperature of 31.9-37.5° C, had one of the three warming devices applied within 30 minutes of arrival, and discharged from the ED to operating room, intensive care unit, or intermediate care unit. Notably, patients were originally required to have an initial core (esophageal, bladder, or rectal) temperature recorded in the ED and be intubated prior to hospital arrival or within 30 minutes after arrival; however, these requirements proved to be highly restrictive and were subsequently removed after data collection had commenced. Patients were excluded if they were pregnant, required the room temperature to be increased for burns, had burn injuries to the posterior side of the body (contra-indication for the forced air underbody warming mattress), or if temperature was not recorded within the first 30 minutes of ED arrival, hourly in the ED, or within 30 minutes of arrival to the post-ED unit.

Data collection and study variables

Temperature data collection involved recording initial ED temperatures within 30 minutes of arrival, temperatures at approximately hours 1-4 after arrival, and temperature on arrival to the post-ED unit. The time and measurement route (i.e., bladder, esophageal, oral, or rectal) of each temperature was recorded. Oral and rectal temperatures were recorded with a standard digital thermometer, SureTemp Plus 692 Thermometer (Welch Allyn, Inc., Skaneateles Falls, New York, United States). Core temperatures were obtained either through an esophageal probe (Level 1 Acoustascope Esophageal Stethoscope with Temperature Sensor Thermistor, Model ES400-12/18; Smiths Medical, Inc., Minneapolis, Minnesota, United States) or a urinary catheter probe (Total One Layer Tray SelectSilicone Temperature Sensing Urinary Catheter; Medline Inc., Northfield, Illinois, United States).

Additional data collection included recording hospital site, age, sex, ED disposition, injury type, injury severity score, height, weight, receipt of warmed fluids, and undergoing any procedures that might interfere with the effectiveness of the warming devices (chest tube, central line, diagnostic peritoneal lavage, focused assessment with sonography in trauma (FAST) exam, urinary catheter insertion, resuscitative endovascular balloon occlusion of the aorta (REBOA) placement, fracture reduction, or splint placement).

The primary outcome variable for this study was the composite outcome of successful thermal management in the full sample, which was defined as achieving normothermia (≥36.5° C) in initially cold stressed (<36.5° C) patients and preventing new onset cold stress (<36.5° C) among initially normothermic (≥36.5° C) patients, both within the first four hours of arrival. Secondary outcomes consisted of separately analyzing the two components of the primary outcome (i.e., achieving normothermia or preventing cold stress in the respective subsets). Due to the study’s setting in the southwestern United States, cold stress was used as the endpoint because the more widely used <35° C definition of hypothermia was considered too rare to use as an outcome.

Statistical analysis

After chart review, five cases (2.4%) were missing height or weight. This small amount of missingness was addressed by using multiple imputation via chained equations to create 10 imputed datasets; the imputed datasets were then collapsed by using the median imputed values of height and weight to create a single dataset with no missingness.

As it is important not only to achieve successful thermal management but to achieve it quickly, primary and secondary outcomes were modeled as time-to-event data right-censored at four hours. The four-hour censoring window was selected to highlight the potential effects of warming devices within the early, acute phase of care, as well as minimize the influence of any later interventions unrelated to initial management in the trauma bay. This was done with Cox proportional hazards models. The models estimated the effects of the warming devices while controlling for arrival temperature, body surface area (Du Bois formula: meters2 = 0.007184 * weight(kg)^0.425^ * height(cm)^0.725^), receiving warmed fluids, and undergoing interfering procedures. Additionally, to control for potential measurement error resulting from the use of different temperature measurement routes, the models adjusted for measurement route at time of event or at censoring, temperature measurement route on arrival, and an interaction of arrival temperature and arrival temperature measurement route. This accounted for both differences in measurement route between different patients as well as potential changes in measurement route within the same patient. Nesting of patients within hospitals and potential confounding effects of the study site were addressed with cluster robust standard errors.

We also performed an exploratory analysis comparing the change in temperature over time among patients treated with the forced air underbody warming mattress at the two sites where it was used. This analysis was conducted upon realizing the two sites differed in when and how the forced air underbody warming mattress was implemented. One site applied the forced air underbody warming mattress on patient arrival; the other applied the warming mattress after initial assessment was performed and the patient returned from the computerized tomography (CT) scanner, but within the 30-minute requirement. This was modeled with a multi-level linear model of patients treated with the forced air underbody warming mattress that allowed intercepts and the slope of time to vary randomly by site, controlled for the same variables as the Cox models, and accounted for nesting of multiple temperature measurements within patients by allowing intercepts to vary randomly as a function of patient.

Finally, the feasibility of the devices was investigated post hoc upon noting substantial differences in the number of patients treated with each warming method. The feasibility investigation was informal and consisted of conducting chart reviews to identify when and why devices were not used, as well as follow-up inquiries to clinical staff about the lack of device use.

Results from the Cox models are summarized with hazard ratios (HRs). Results from the linear model are summarized with the predicted change in temperature from arrival, with covariates set at their mean or mode. Model diagnostics were assessed via examination of variance inflation factors, residuals, and score tests of the proportional hazards assumption. All data were analyzed in R version 4.4.0 (R Core Team, Vienna, Austria). Additional packages essential to analysis included tidyverse for data management, mice for multiple imputation, and survival and lme4 for model fitting [18-21].

## Results

**Figure 1:**
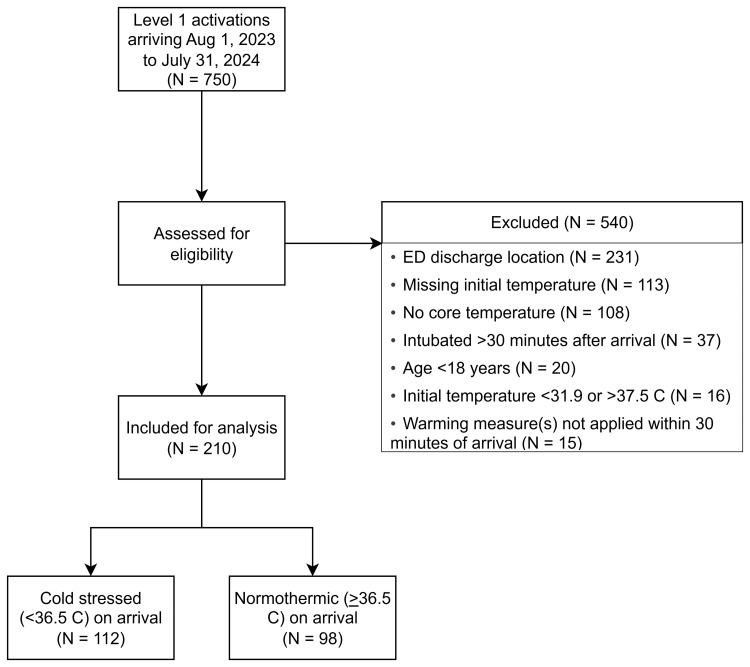
Study Flow Chart

**Figure 2:**
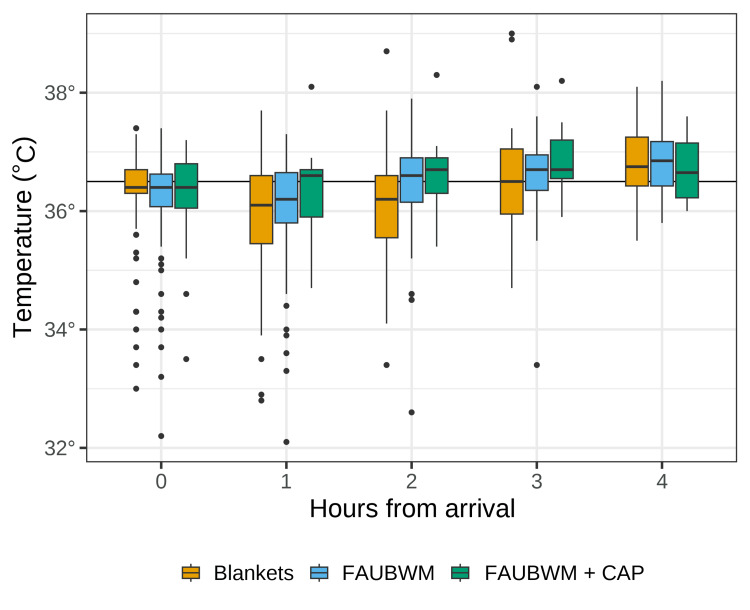
Patient temperatures by warming method and hour Horizonal line at 36.5° C represents the threshold for cold stress. Temperatures are not adjusted for confounders. CAP: thermo-reflective cap; FAUBWM: forced air underbody warming mattress

A total of 750 patients were screened. Of these, 540 were excluded, primarily for ED discharge location (231, 42.7%) (Figure 1). This left a final analysis sample of 210 patients. Table 1 shows the characteristics of the study population. The sample was primarily male (72.4%) with a mean ± SD age of 45.9 ± 19.3 years and mostly blunt injuries (76.7%). The mean arrival temperature was 36.21 ± 0.89, with 112 (53.3%) patients classified as cold stressed on arrival. The majority of patients were seen at the first Level I trauma center (150, 71.4%), with 15 (7.1%) treated at the second Level I center, and 45 (21.4%) treated at the Level II center. A slight plurality of patients were treated with blankets (99, 47.1%), with slightly fewer treated with forced air underbody warming mattress (92, 43.9%). Only 19 patients (9.0%) received the forced air underbody warming mattress + cap due to the prevalence of contraindicated head or scalp injuries in this population; results involving this group should be interpreted with caution due to its small sample size. Figure 2 shows the temperatures for each warming method across the four hours of observation. In addition to the warming methods under investigation, 58.6% of patients received warmed IV fluids or blood, with notable variability between treatment groups as shown in Table 1.

**Table 1:** Characteristics of the study population (N=210) ^a^ Precise measurement route not documented, only “core temperature” ED: emergency department; ICU: intensive care unit; FAUBWM: forced air underbody warming mattress.

Variable	Overall	Blankets	FAUBWM	FAUBWM+Cap
Frequency, n (%)	210 (100%)	99 (47.1%)	92 (43.9%)	19 (9.0%)
Level I trauma center A, n (%)	150 (71.4%)	72 (72.7%)	59 (64.1%)	19 (100.0%)
Level I trauma center B, n (%)	15 (7.1%)	15 (15.2%)	0 (0.0%)	0 (0.0%)
Level II trauma center, n (%)	45 (21.4%)	12 (12.1%)	33 (35.9%)	0 (0.0%)
Blunt injury, n (%)	61 (76.7%)	72 (72.7%)	75 (81.5%)	14 (73.7%)
Age, years, mean ± SD	45.9 ± 19.3	44.4 ± 19.2	46.9 ± 19.0	48.0 ± 21.1
Male sex, n (%)	152 (72.4%)	75 (75.8%)	62 (67.4%)	15 (78.9%)
ED discharge to ICU/step-down, n (%)	155 (73.8%)	69 (69.7%)	71 (77.2%)	15 (78.9%)
Injury severity score, mean ± SD	17.5 ± 12.4	19.4 ± 13.5	16.3 ± 11.3	14.1 ± 9.9
Body surface area, m^2^, mean ± SD	1.99 ± 0.25	2.00 ± 0.24	1.96 ± 0.26	2.04 ± 0.23
Received warmed fluids, n (%)	123 (58.6%)	52 (52.5%)	57 (62.0%)	14 (73.7%)
Any interfering procedure, n (%)	180 (85.7%)	87 (87.9%)	77 (83.7%)	16 (84.2%)
Normothermic (≥36.5) on arrival, n (%)	98 (46.7%)	48 (48.5%)	41 (44.6%)	9 (47.4%)
Cold stressed (<36.5) on arrival, n (%)	112 (53.3%)	51 (51.5%)	51 (55.4%)	10 (52.6%)
Successful thermal management within 4 hours, n (%)	89 (42.4%)	34 (34.3%)	47 (51.1%)	8 (42.1%)
Temperatures, C, mean ± SD				
On arrival	36.21 ± 0.89	36.24 ± 0.87	36.17 ± 0.91	36.22 ± 0.94
At hour 1	36.04 (1.02)	35.94 (1.13)	36.05 (0.95)	36.35 (0.78)
At hour 2	36.26 (1.02)	36.02 (1.13)	36.38 (0.94)	36.66 (0.83)
At hour 3	36.62 (0.94)	36.54 (1.06)	36.60 (0.88)	36.90 (0.75)
At hour 4	36.81 (0.60)	36.82 (0.68)	36.82 (0.52)	36.72 (0.72)
On post-ED unit arrival	36.22 (1.09)	36.00 (1.13)	36.40 (1.04)	36.55 (0.90)
Temperature measurement route on arrival, n (%)				
Bladder	19 (9.0%)	12 (12.1%)	5 (5.4%)	2 (10.5%)
Esophageal	9 (4.3%)	5 (5.1%)	4 (4.3%)	0 (0.0%)
Oral	122 (58.1%)	61 (61.6%)	48 (52.2%)	13 (68.4%)
Other core^a^	11 (5.2%)	6 (6.1%)	4 (4.3%)	1 (5.3%)
Rectal	49 (23.3%)	15 (15.2%)	31 (33.7%)	3 (15.8%)

Achieving successful thermal management

**Figure 3:**
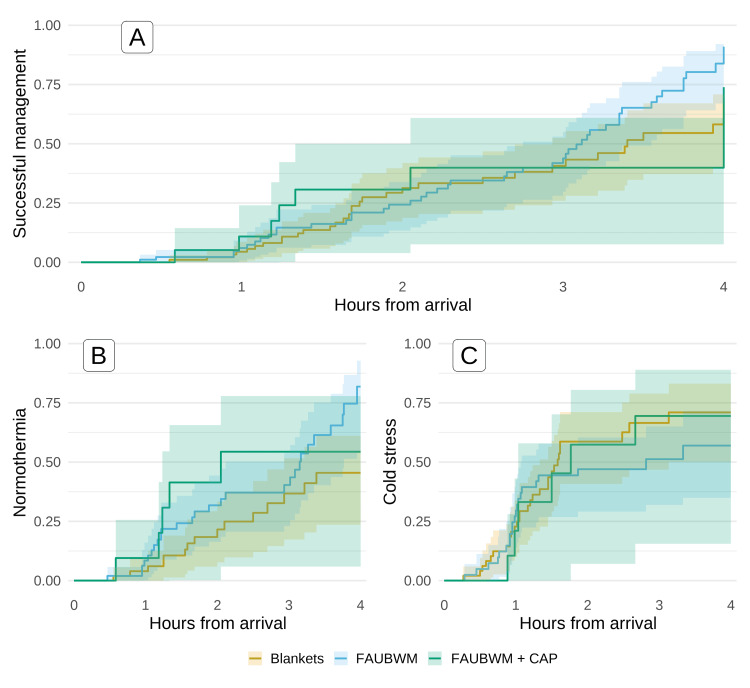
Kaplan-Meier curves of primary and secondary outcomes A: Probability of successful thermal management in the full sample; B: Probability of achieving normothermia (≥36.5° C) among patients who were initially cold stressed (<36.5° C); C: Probability of developing cold stress (<36.5° C) among initially normothermic patients (≥36.5° C). Shaded areas represent cluster-robust 95%CIs.  Estimates are not adjusted for confounders. CAP: thermo-reflective cap; FAUBWM: forced air underbody warming mattress

Mean arrival temperatures and proportions of patients in cold stress on arrival were nearly identical across the treatment groups. Within four hours of arrival, 34 (34.3%) patients treated with blankets, 47 (51.1%) patients treated with forced air underbody warming mattress, and eight (42.1%) patients treated with forced air underbody warming mattress + cap had successful thermal management. The unadjusted mean time to successful thermal management was 3.0 hours for blankets, 2.8 hours for forced air underbody warming mattress, and 2.9 hours for forced air underbody warming mattress + cap (Figure 3A).

Unadjusted estimates from the Cox model showed the forced air underbody warming mattress with or without a cap did not significantly differ from blankets (Table 2). The adjusted Cox model showed similar results, with the forced air underbody warming mattress being 58% more effective but not significantly different than blankets (p = 0.104). Using forced air underbody warming mattress + cap appeared slightly worse than but not significantly different from blankets (p = 0.293).

**Table 2:** Summary of models examining therapeutic effectiveness of warming devices For successful management and achieving normothermia, HR >1 indicates benefit.  For preventing cold stress, HR <1 indicates benefit.  Unadjusted models control for nesting of patients within hospitals.  Adjusted models controlled for arrival temperature, measurement routes, body surface area, receiving warmed fluids, undergoing interfering procedures, and nesting of patients within hospitals. HR: hazard ratio; FAUBWM: forced air underbody warming mattress.

Warming Device	Successful management			Achieving normothermia			Preventing cold stress		
	Unadjusted HR (95% CI)	Adjusted HR (95% CI)	P-value	Unadjusted HR (95% CI)	Adjusted HR (95% CI)	P-value	Unadjusted HR (95% CI)	Adjusted HR (95% CI)	P-value
Blankets (referent)	1.00	1.00	-	1.00	1.00	-	1.00	1.00	-
FAUBWM	1.50 (0.57 – 3.95)	1.58 (0.91 – 2.76)	0.104	1.95 (0.97 – 3.91)	4.74 (3.55 – 6.34)	<0.001	0.77 (0.59 – 1.00)	0.77 (0.44 – 1.33)	0.343
FAUBWM + cap	0.82 (0.50 –1.35)	0.74 (0.42 – 1.30)	0.293	1.18 (0.61 – 2.26)	2.23 (0.80 – 6.25)	0.127	1.30 (0.00 – 1.69)	2.24 (2.05 – 2.43)	<0.001

Treating initially cold-stressed patients

There were 112 (53.3%) patients with cold stress (<36.5° C) on arrival. Of these, 51 (45.5%) were treated with blankets, 51 (45.5%) with forced air underbody warming mattress, and 10 (8.9%) with forced air underbody warming mattress + cap. The mean arrival temperature was 35.7 ± 0.9, and 47 (42.0%) patients achieved normothermia within four hours. As demonstrated in Figure 3B, the unadjusted average time to normothermia was 3.1 hours for blankets, 2.8 hours for forced air underbody warming mattress, and 2.5 hours for forced air underbody warming mattress + cap. The adjusted Cox model revealed that compared to blankets, treatment with a forced air underbody warming mattress was associated with a nearly five-fold increase in achieving normothermia (HR = 4.75, p < 0.001). Use of forced air underbody warming mattress + cap was associated with a non-significantly increased risk of achieving normothermia relative to blankets (HR = 2.23, p = 0.127).

Preventing new cold stress

There were 98 (46.7%) patients who were normothermic (>36.5° C) on arrival. Of these, 48 (49.0%) were treated with blankets, 41 (41.8%) with forced air underbody warming mattress, and 9 (9.2%) with forced air underbody warming mattress + cap. The mean arrival temperature was 36.8 ± 0.3, and 57.1% became cold stressed within four hours. As shown in Figure 3C, the unadjusted average time to cold stress was 2.1 hours for blankets, 2.5 hours for forced air underbody warming mattress, and 2.1 hours for forced air underbody warming mattress + cap. The adjusted Cox model showed that compared to blankets, treatment with forced air underbody warming mattress was associated with a nonsignificant reduction in cold stress risk (hazard ratio (HR) = 0.77, p = 0.343). Inversely, forced air underbody warming mattress + cap was associated with a significant 124% increased risk of cold stress relative to blankets (HR = 2.24, p < 0.001).

Comparison of forced air underbody warming mattress at two sites

**Figure 4:**
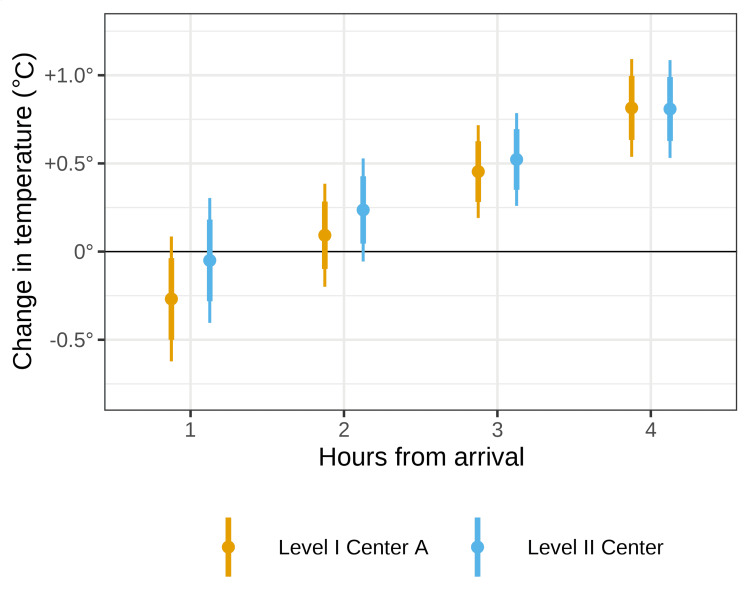
Hourly changes in temperature among patients treated with forced air underbody warming mattress by site Points represent the predicted mean temperature change.  Thick lines represent the 80% confidence interval of the mean.  Thin lines represent the 95% confidence interval of the mean.  Predictions adjusted for arrival temperature, measurement routes, body surface area, receiving warmed fluids, undergoing interfering procedures, nesting of patients within hospitals, and nesting of repeated observations within patients.

Among the 92 patients treated with a forced air underbody warming mattress, 59 were treated at level I trauma center A (arrival temperature 35.95 ± 1.11) and 33 at the level II center (arrival temperature 36.47 ± 0.56). A test of the random slope of time by site showed patients did not warm at significantly different rates at the two hospitals (p = 0.764). Despite this test of the overall trend, at one hour after arrival, patients at the level II center that applied the underbody warming mattress on arrival were predicted to have an average temperature change since arrival of -0.05° (95%CI = -0.40 to +0.30) versus -0.27° (95%CI = -0.62 to +0.09) at the level I center that applied the underbody mattress after assessment and returning from CT. However, as shown in Figure 4, the difference between sites decreased over the next three hours such that by four hours, patients at both sites were predicted to have warmed by +0.81° (95%CI = +0.54 to +1.09).

## Discussion

The need for temperature management in trauma is well documented [3,22-25]. Despite this, there is sparse literature examining methods to treat and prevent cold stress or hypothermia in the trauma bay, with no well-established method for temperature management identified. This multicenter study attempted to address this gap by comparing the effectiveness of blankets, forced air underbody warming mattress, and forced air underbody warming mattress + cap as methods to treat and prevent cold stress. One of the first findings from this study was the apparent infeasibility of the thermo-reflective cap in this population due to the high prevalence of contraindicating head wounds, which resulted in only 19 patients (9.0%) receiving forced air underbody warming mattress + cap application. In contrast, the forced air underbody warming mattress appears to be substantially more effective than blankets at treating existing cold stress; however, it was not reliably better than blankets for overall thermal management or prevention of new onset cold stress.

The relative lack of research into ED temperature management methods may partially be due to a lack of awareness of the forced air underbody warming mattress. The forced air underbody warming mattress has clear appeal as a possible method to warm half of the body while ensuring access to the patient. As this study shows, its theoretical benefit appears to manifest in practice.

Evaluating the practicality of the warming methods was as important as evaluating effectiveness. Means to prevent cold stress hypothermia are available, but they will not be adopted if they are not practical in complex trauma resuscitation. Overbody warming mattress is not feasible in trauma due to the cumbersome raft-like mattress obstructing access to the patient [26]. Likewise, the practicality of the overhead cap was not feasible for full trauma activations because many patients had head injuries. The protocol called for no cap application if the patient experienced head trauma. The neurosurgeon (involved from the beginning of the project) approved and was in favor of the cap application. The neurosurgeon’s concern was the cap possibly being placed prior to assessment, which could potentially obscure head injuries such as scalp lacerations. In addition to this possibility, the caps have an outer metal film that interferes with CT scans, meaning the caps would have to be removed and then later replaced for CTs to be completed. These additional logistical hurdles made routine use of the cap impractical.

When the forced air underbody warming mattress was compared by site, the level II trauma center experienced notably less heat loss in the first hour than the level I center due to applying the underbody mattress sooner. Upon investigating why, the level II center places its warming machine under the boom at the head of the stretcher, connects the machine to the forced air underbody warming mattress prior to arrival, and continues its use while CTs are performed. In the level I center, the machine was placed at the end of the stretcher, where the cord was consistently in the way, especially with large numbers of people at the bedside. Additionally, the patient was assessed, treated, completed CT scans, and then placed on the forced air underbody warming mattress when returning from CT (but within 30 minutes of arrival). 

The above procedural differences may also help to explain the counterintuitive finding that the forced air underbody warming mattress with a cap was associated with a significantly higher risk of developing new cold stress relative to blankets. Since the use of the warming mattress was associated with a nonsignificantly lower risk of new cold stress when compared to blankets, it seems unlikely that the addition of the thermo-reflective cap would inherently result in worse outcomes. Despite the statistically significant result, the potential for small sample bias to explain this finding should also not be overlooked, since it is based on only nine patients treated with the thermo-reflective cap. 

Limitations

Although the results of this study support the use of a forced air underbody warming mattress over blankets to treat existing cold stress, it is subject to multiple limitations. The ambient room temperature at each site was not regulated or controlled for. But the authors argue that in clinical practice, maintaining the room temperature at a certain degree is impractical, especially if the rooms are used for multiple types of high-risk patients. As a retrospective cohort study, we are unable to establish causality, and therefore, all findings are only observational in nature. Despite rigorous statistical adjustment, we cannot eliminate the potential for bias or confounding to affect the results, including potential measurement error resulting from the use of different temperature measurement routes. It is also possible that some patients treated with a warming mattress were also treated with blankets, which could bias results towards the null. Ideally, patients would have temperatures assessed with the same thermometers within the same timeframes, and workflows would be consistent across study sites; however, this degree of standardization is frequently impractical in trauma bays where individual injury patterns, changes in levels of consciousness, or procedures can necessitate using different measurement routes. Additionally, while workflow and measurement heterogeneity do negatively impact internal validity, they can increase generalizability; this tradeoff is inherent to the decision to conduct effectiveness research (i.e., what tends to happen in real-world, practical settings) versus efficacy research (i.e., what can happen in ideal, tightly controlled settings). Patients treated with forced air underbody warming mattresses with or without the cap may meaningfully differ from those treated with blankets in ways that we were unable to account for due to unobserved confounders. These points would ideally be addressed with a prospective study. 

Additionally, although the forced air underbody warming mattress significantly outperformed blankets as a method to treat cold stress, the study’s setting in the southwestern United States means that severe hypothermia is rare. Indeed, even among patients classified as cold stressed on arrival, the mean temperature was 35.7 °C. This, plus the study’s relatively restrictive inclusion criteria, may limit the generalizability of the results. Changing the study’s inclusion criteria after initiation to address slower than anticipated accrual may also have influenced the results in unknown ways by introducing selection bias and hindering replicability. The study also suffered from a smaller sample size than desired based on power analysis; it is plausible that the forced air underbody warming mattress is superior to blankets for overall thermal management and for preventing cold stress, but this study was underpowered to detect such effects. The very limited number of patients with thermo-reflective head caps precludes any meaningful insights for this device. Finally, although the deleterious effects of hypothermia are well-established in the trauma literature, this study did not investigate any longer-term clinical implications of the warming devices; it is unclear if the temperature differences that were observed with the warming devices resulted in clinically meaningful changes (e.g., on length of stay, transfusion requirements, or morbidity and mortality).

## Conclusions

Although the negative effects of hypothermia in trauma are well-documented, there has been little research into cold stress and temperature management in the trauma bay. This multicenter study compared the practicality and effectiveness of blanket application versus a forced air underbody warming mattress with and without a thermally reflective head cap. The cap was found to be impractical in the setting of prevalent head injuries and the routine need for CT scans. Conversely, the underbody warming mattress appears to be both practical in the trauma bay and more than four times more effective than blankets at treating existing cold stress.

Based on these results, the participating centers are planning to increase the use of the underbody warming mattress in the ED. The change will be based on both the thermal outcomes of the study and the ease of use in clinical practice. The mattress can be placed on the stretcher prior to the patient’s arrival. Once the patient arrives, the machine can be attached to the mattress located at the head of the bed. This practice flow change encountered minimal resistance from the staff and was considered easily achievable. Although this nursing intervention is anticipated to reduce hypothermia, additional research is needed to establish best practices for temperature management in the trauma bay.
